# Critical View on Dielectric Constant Changes in Solid and Liquid Nonanol

**DOI:** 10.1021/acs.jpclett.6c02749

**Published:** 2026-10-27

**Authors:** Aleksandra Drozd-Rzoska, Sylwester J. Rzoska, Aleksander Szpakiewicz-Szatan

**Affiliations:** 49559 Institute of High Pressure Physics of the Polish Academy of Sciences, ul. Sokołowska 29/37, 01-142 Warsaw, Poland

## Abstract

The report presents evidence for the long-range premelting effect, with a well-defined critical-like scaling pattern, in the dielectric constant (*ε*) of solid nonanol, extending even ∼40 K below the melting temperature *T*_*m*_, namely, *ε*(*T*)∝1/(T* – *T*), the singular temperature T* ≈ *T*_*m*_ + 0.4 K. This is supplemented by tests in liquid nonanal showing exceptional evidence for a simple scaling relation *ε*(*T*) = *a* + *b*/*T*, *a*,*b* = *const*. The experiment also revealed unique Mossotti Catastrophe type behavior in liquid nanolayers between crystalline grains in the premelting domain. It can be associated with rising negative pressure, finally leading to abrupt implosive ultragranulation and liquidation at *T*_*m*_. For the applied experimental method, the response of quasi-liquid nanolayers is qualitatively greater than that from crystalline grains in the premelting region. This is consistent with the Lipowsky model [ Phys. Rev. Lett. 1982, 49, 1575 ], suggesting the criticality of nanoconstrained quasi-liquid layers between solid plates.

## Graphical abstract

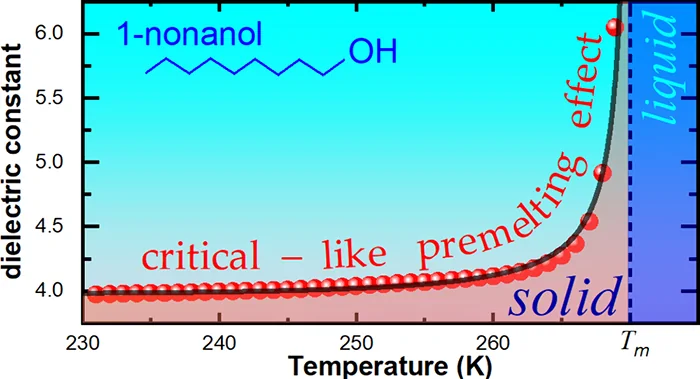

Understanding and modeling continuous/critical phase transitions are among the grand successes of 20th-century physics.^1,2^ The key inspirations were long-range pretransitional effects, described by power-type scaling relations with critical exponents that depend solely on the space (*D*) and order-parameter (*n*) dimensionalities. This extraordinary universalism, which limits the role of systems’ microscopic features, results from the dominant influence of multimolecular critical fluctuations, whose lifetime (τ_*fl*._) and size (ξ, correlation length) increase infinitely on approaching the critical temperature:^2,3^

1 $$\xi \left(T\right)\propto {\left|T-T_{C}\right|}^{-\nu },\tau_{fl.}\left(T\right)\propto {\left|T-T_{C}\right|}^{z\nu }$$

where ν is the correlation length exponent: ν = 1/2 for the mean-field approximation obeying for *D* > 4; and *z* is the dynamic exponent: *z* = 2 for the nonconserved order parameter, obeying for *D* > 4, and *z* = 3 is for the conserved one.

Recalling continuous, critical phase transitions highlights the conceptual and cognitive contrast with the discontinuous melting transition, which is the topic of this report. Equally important is this report’s finding: critical behavior can be a “hidden, driving phenomenon” behind the melting transition.

Discontinuous transitions, remains a cognitive puzzle despite more than a century of study.^3–5^ The canonical example is the melting transition between the solid crystal and liquid. The recent topical review by de With^5^ shows the puzzling state of the art, with a pessimistic final conclusion:^5^ “*it may well be that a generally applicable mechanistic theory, in view of the many aspects that require attention, does not exist*”.

This qualitative cognitive distinction between continuous and discontinuous transitions can be linked to the specific manifestation of discontinuous transitions and the virtual absence of pretransitional effects, which are the “conceptual reference” for continuous phase transitions.

The still-dominant framework for the discontinuous phase transitions, particularly the melting, was proposed by Clapeyron and Clausius (1850/1870)^6,7^ and by Lindemann (1910).^8^ The Clausius-Clapeyron (C-C) equation addresses the basic manifestation, namely, step changes in volume (Δ*S*) and enthalpy (Δ*H*) or heat capacity on passing the melting temperature *T*_*m*_:^6,7^

2 $$\frac{dT_{m}}{dp}=T_{m}\frac{\Delta V}{\Delta H}=\frac{\Delta V}{\Delta H/T_{m}}=\frac{\Delta V}{\Delta S}$$

where *p* denotes pressure, *ΔS* = *L*/*T*_*m*_ is for the entropy change and d*T*_*m*_/d*p* describes the pressure (*p*) impact on the melting temperature *T*_*m*_.

Lindemann^8^ developed the model concept linking melting to thermal vibrations of atoms about their positions within the crystalline solid. Above a certain temperature *T*_*M*_ < *T*_*m*_ the ratio between the amplitude of these vibrations and the interatomic spacing can become too large for the solid to remain ordered, and then melting can take place. This was expressed by the following equation:^5,8^

3 $$\frac{dT_{m}}{dp}=2T_{m}\left(\gamma_{G}-\frac{1}{3}\right)B^{-1}$$

where *B* = (−*V* d*p*/d*V*)_*T*→*T*_*m*__ is the bulk modulus reflecting the system’s resistance to compression (pressure) and γ_*G*_ means the Grüneisen parameter addressing network vibrations.

For decades, melting transition research has referred to the above relations, supported by spectroscopic and structural studies of the solid crystal side of *T*_*m*_.^4–7^

Notwithstanding, when approaching *T*_*m*_ a premelting effect resembling a specific pretransitional anomaly can occur, although the experimental evidence remains puzzling. First, it is not present in all systems, nor for all physical properties.^9–18^ Second, the manifestations of premelting effects are so diverse that their general temperature-related scaling pattern is not considered, even in model analyses. Nevertheless, premelting effects are regarded as essential for understanding the nature of melting.^9–18^

Several models address this issue.^10–18^ The so-called grain model, which links the phenomenon to the formation of solid crystalline grains surrounded by quasi-liquid nanolayers, is particularly important to the authors. The advanced experimental evidence demonstrating the existence of such solid crystalline grains in the premelting region is worth emphasizing.^13,15–18^

Notably, it is unclear which interfaces trigger the onset of premelting and facilitate its development.^13,15–18^ The question of how grain size changes as *T*_*m*_ is approached is still unclear. Consequently, the mechanism of premelting, particularly its temperature dependence, remains a research challenge.

Among concepts related to the premelting grain, the model by Lipowsky^19,20^ is exceptional because it includes explicit temperature-related output-scaling equations. Lipowsky considered the behavior of a liquid constrained in a nanolayer between solid plates, related to the quasi-liquid in nanolayers between solid grains. Using the Landau model, he derived several scaling relations. For this report, particularly important is that related to the compressibility χ_*T*_:^19,20^

4 $$\chi_{T}\left(T\right)=\left(1/V\right)\times dV/dp={\left\lceil T^{*}-T\right\rceil }^{{-\gamma }_{s}}$$

where for the susceptibility exponent γ_*S*_ ≈ 1, although in some cases when the layer thickens rises the layer: γ_*S*_ = 1 – 2β_*l*_. with the order parameter exponent β_*l*_.

The key problem with experimentally validating the Lipowsky model is that the volume occupied by the quasi-liquid nanolayer is qualitatively smaller than that of solid crystalline grains. Most physical methods monitor the response of the entire sample volume, making experimental validation practically impossible.^19,20^ This is probably why the Lipowsky model has almost disappeared from research discussions on the melting transition over the last quarter-century,^3–5,10–18^ despite its analytical beauty.^19,20^

This report presents evidence of the long-range pretransitional/premelting effect in the real part of the dielectric permittivity of solid 1-nonanol, focusing on the dielectric constant. Using this method, the response from the quasi-liquid phase is essentially larger in magnitude than that from the solid grains. This enables direct, high-resolution insight into their properties. Studies revealed a critical pattern attributed to a Mossotti Catastrophe,^21–23^ which also links to the Lipowsky model.^19,20^ These findings suggest a new model of premelting behavior and melting transition, in which a discontinuous transition is driven by a continuous one.

Studies of 1-nonanol in the liquid phase have revealed an unexpected pattern of temperature-dependent changes in the dielectric constant. 1-Nonanol (hereafter referred to as “nonanol”) is a canonical material in the fields of *Dielectric Physics* ^21–25^ and *Physical Chemistry*.^26,27^ It is a carbon straight-chain aliphatic alcohol, (CH_3_(CH_2_)_7_CH_2_OH), with the permanent dipole moment μ ≈ 1.6 D.^22^ Studies were carried out using a Novocontrol Alpha impedance analyzer and focused on the real part of dielectric permittivity. Tests were carried out at 150 temperatures in the range from 140 to 330 K, with 5–6 digits resolution. They were supported by an extended Quattro temperature control unit, resolution from ±0.02 to ±0.1 K. Tested samples were placed in a flat-parallel capacitor, made from gold-coated Invar: diameter 2*r* = 20 mm and distance between plates *d* = 0.3 mm. A quartz ring was used as a spacer. Nonanol was purchased from Fluka at the highest available purity class and degassed prior to measurements.

Figure 1 presents the experimental spectra of the real and imaginary parts of the dielectric permittivity at selected temperatures relevant to this report. This report focuses on the real part, where the horizontal domain determines the dielectric constant *ε*, for which *ε*′(*f*) = *ε* = *const*, for arbitrary frequency. For lower frequencies (LF domain), the dielectric permittivity permanently increases, which is linked to the translational motion of “ionic impurities”,^24,25^ but the translational motion of basic molecules in the system may be equally important. The plot also shows a significant change in dielectric constant when passing from the solid to the liquid phase, which is particularly significant to the subject of this report. All dielectric constant values were taken in the middle of the static domain.

**Figure 1:**
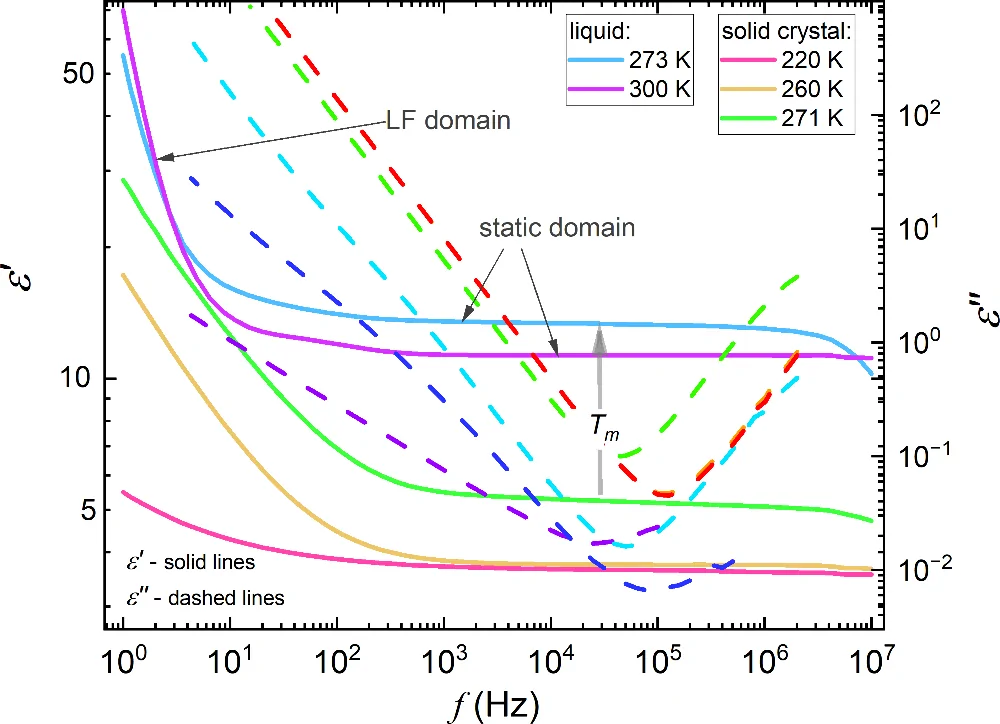
Illustration of the spectrum of the real part of dielectric permittivity in the liquid and solid phases of ortho-nitrotoluene, whose structure is shown in the plot. For the static domain, the frequency shift does not change the value of dielectric permittivity, which defines the dielectric constant, i.e., *ε*′(*f*) = *ε* = *const*; in the low-frequency (LF) domain a strong rise occurs.

Dielectric constant behavior in liquid dielectrics is modeled employing the assumption that the field acting on the molecule inside differs from the external electric field. This led to the local field concept:^21–25^ *F* = *E* + *E*_1_ + *E*_2_, where *E*_1_ is from the surface charge of a cavity surrounding a symbolic molecule, and *E*_2_ is for the electric field within the cavity. The basic model description, commonly used for the analysis of experimental data, is the relation^22,24,25^

ε−ε∞=L(ε,ε∞)Nμ23εokBTgK(5a)⇒ε−ε∞=Nμ23εokBT(5b)

where the right is for model descriptions obeying in dielectric liquids and the right-hand part describes the behavior in boundary conditions of a dipolar component strongly diluted in a neutral, nondipolar solvent or dipolar molecular systems in gaseous state for which *ε* – *ε*_*∞*_ ≪ 1; *ε*_*∞*_ is for nondipolar contribution related to atomic and molecular polarizabilities, *N* is the number of permanent dipole moments in a unit volume, *k*_*B*_ is the Boltzmann constant, *L* is for the local field factor, and *g*_*K*_ denotes Kirkwood dipole–dipole correlation factor.

For the most often implemented Kirkwood local field approximation:^22,24,25^ *L* = 3*ε*/[(2*ε* + *ε*_∞_)­((*ε*_∞_ + 2)/3)^2^], and the dipole–dipole correlation factor *g*_*K*_ = 1 + *z* cos θ. When thermal motion energy is significantly higher than the energy leading to dipole–dipole coupling: *z* cos θ → 0 an *g*_*K*_ → 1 and eq 5a reduces to the basic reference Onsager local field model.^22^ For the latter, the cavity is considered comparable to molecular size, but close-range interactions are small when compared with *k*_*B*_ *T*, expected for gases, with no significant local field.^21,22^

Figure 2 shows a high-resolution scan of the dielectric constant over a wide temperature range (not reported so far), covering both the liquid and solid states. The behavior of the dielectric constant in molecular liquids is most often discussed within eq 5a. However, it does not enable the scaling of dielectric constant temperature changes *ε*(*T*). Its implementation focuses on concentration (*x*) related studies in nondipolar solvents, because of the inherent *N*(*x*) dependence or on testing the Kirkwood factor *g*_*K*_(*x*, *T*) evolution.^25–34^

**Figure 2:**
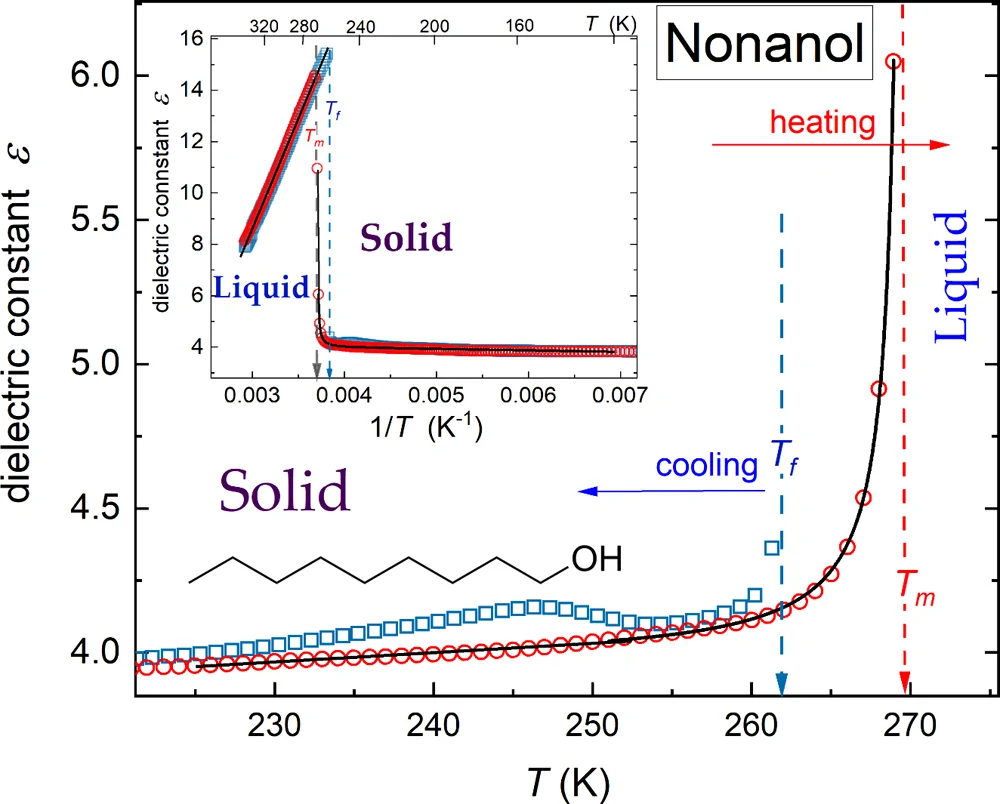
Temperature changes of the dielectric constant on heating from the solid phase, via the melting temperature *T*_*m*_, and on cooling from the liquid phase via the freezing temperature *T*_*f*_.

The inset in Figure 2 reveals effective scaling of temperature changes: *ε*(*T*) = *a* + *b*/*T* in the liquid state, resembling eq 5b which is generally considered only for gaseous systems.^22^ This is definitely not the case of liquid nonanol. Notably, dielectric studies in nonanol and other monohydric alcohols are included in fundamentals of *Physical Chemistry*, due to the illustration of insight into molecular structure and hydrogen bonding, which also dictate self-association into multimers. The latter is linked to *g*_*K*_ > 1.^22,25–34^

The authors’ working hypothesis is that this exceptional pattern of *ε*(*T*) changes is related to the dominance of multimolecular associates, which may yield an effective dipole moment matched with weak mutual interactions. Nevertheless, this result indicates the need for a revised view of the dielectric properties of nonanol and other monohydric alcohols in the liquid state.

The central part of Figure 2 focuses on the premelting effect in solid nonanol for *T* → *T*_*m*_. It is scaled fairly by the solid curve shown in Figure 2, parametrized by a critical-like relation:

6 $$\varepsilon^{\prime }\left(T\right)={\left(a+bT\right)}_{bckg}+\frac{A}{{\left(T-T^{*}\right)}^{\gamma }},,\gamma =1$$

where “*bckg*” denotes the background term, dominated remote from *T*_*m*_: *a* = 3.354, and *b* = 0.027 (K^–1^) are related parameters for nonsingular linear behavior *T* = 220 K; *A* = 1.510 K^–1^ is the amplitude for the “critical” premelting anomaly, and T* = *T*_*m*_ + 0.4 K is the extrapolated singular, critical-like temperature, *T*_*m*_ = 269.70 K.

Note: the singularity in eq 6, i.e., T*, lies slightly above the discontinuous melting transition temperature *T*_*m*_, in agreement with the preliminary model discussion presented below and the Lipowsky model.^19,20^

It is notably, that eq 6 resembles the Mossotti Catastrophe pattern,^21–23,35,36^ which is the direct consequence of Clausius-Mossotti (C-M) local-field model,^21,22,35,36^ the first and simplest one (1850/1876).^35,36^ For this model a semimacroscopic cavity with negligible short-range intermolecular interactions is considered, i.e., *E*_2_ = 0, and an averaged impact of the remoted surrounding: *E*_1_ = *P*/3*ε*_0_, where *P* means electric polarization and *ε*_0_ = 8.854 pF/m is for vacuum dielectric permittivity. It yields^21–23,35,36^ *F* = [(χ + 3)/3]*E* for the local electric field effectively acting on a symbolic molecule. Taking into account that the electric polarization *P* = *NαF*, where α means molecular polarizability, one obtains^21–23^

7 $$\chi =\varepsilon -1=\frac{P}{\varepsilon_{0}E}=\frac{N\alpha /\varepsilon_{0}}{1-N\alpha /3\varepsilon_{0}}=\frac{3T_{C}}{1-T_{C}}\Rightarrow \chi \left(T\right)=\frac{A}{{\left(T-T_{C}\right)}^{\gamma }},\mathrm{with}\;\gamma =1$$

where the singular temperature *T*_*C*_ = *Nα*/3*ε*_0_.

Von Hippel^21^ implemented the above for water, where the permanent dipole moment contribution (μ ≈ 1.9 D) is dominant, and recalling Debye approximation for dipolar polarizability obtained and obtained: *T*_*C*_ ≈ *N*μ^2^/9*ε*_0_ *k*_*B*_ ≈ 1520 K, which led the statement:^21^ “*Hence water should solidify by spontaneous polarization at high temperature, making life impossible on this Earth*!” Notably that approximately the same *T*_*C*_ “paradox-value” can be estimated for nonanol.

The consistency with experimental results appears for eq 5a, where the model relation includes short-range interactions.

Generally, ferroelectrics are an example of materials where C-M model obeys. This can be linked to a specific way in which dipole moments are created: by shifting charges in certain parts of the crystal lattice.^21,23,25,37^ The inherent uniaxiality of dipole moments created in this way, balanced by translational ordering, may also be of fundamental importance here.^38^ All these lead to the Curie–Weiss relation in the paraelectric phase,^21,23,25,37,38^ in agreement with the pattern given in eq 7.

The C-M model and the Mossotti Catastrophe behavior are “forbidden” for molecular liquids.^21–25^ However, Figure 2 shows an explicit appearance of such behavior in the premelting effect domain. It can be linked to nonanol quasi-liquid nanolayers constrained by solid grain surfaces. This exceptional behavior starts even from *T*_*M*_ ∼ *T*_*m*_ – 40 K.

This may occur because nanoconstraints from solid grain surfaces can lead to a spontaneous preference for parallel molecular alignment, or a uniaxial arrangement of nonanol molecules. This resembles the situation in liquid-crystalline mesophases as already suggested by Lipowsky.^19,20^ Compared with the standard spherical molecules in 3D space, such a uniaxial arrangement increases the number of surrounding molecules that can interact with a given molecule.^38^ This can be interpreted as the manifestation of quasi-spaces with *D* > 4 dimensionalities or equivalently “infinite” range of interactions. Both conditions are essential for the mean-field approximation in *Critical Phenomena Physics*.^1,2^

Nanoconstraints can also create significant negative pressure^39^ for the “trapped” quasi-liquid, constrained in nanolayers. Studies of dielectric relaxation in glass-forming systems explicitly evidenced that under nanoconstraints quasi-negative pressure reaching even *P* ≈ −200 MPa can appear.^40^ The same can be expected for premelting in nonanol, given evidence of a significant decrease in melting temperature in the silicon nanochannel,^41^ whereas compression increases *T*_*m*_. Negative pressure means stretching, i.e., increased intermolecular distances.^39^ This set of factors favors the conditions relevant for the Clausius-Mossotti local-field model. As noted above, this can lead to a mean-field model in which averaged long-range interactions among symbolic molecules lead to Curie–Weiss or Mossotti Catastrophe behavior. Recalling the reference case of paraelectric phase, this means critical-like changes for susceptibility χ = 1 – *ε* = d*P*/d*E* ∝ 1/(*T* – *T*_*C*_), where *P* denotes electric polarization and *E* the electric field, coinciding with eq 7.^1,37,38^ This the Curie–Weiss law, most often derived via the Landau model for continuous phase transitions by considering order-parameter related expansion of the free energy.^1,38^ However, the Landau model considers susceptibility as the derivative of the order parameter with respect to the coupled field – for paraelectrics, namely *P* and *E*, respectively.^1,38^ For standard liquids, the order parameter related susceptibility means compressibility χ_*T*_ ∝ d­(*V* – *V*_*C*_)/d*P* = d*V*/d*p*, where *V* is volume and *p* means pressure.^1^

This report suggests that for a quasi-liquid nanolayer, under unique conditions created under nanoconstraints: χ_*T*_ → χ = *ε* – 1. This also means that the result shown in Figure 2 and eq 6 can be considered an experimental validation of the Lipowsky model,^19,20^ in particular.

The pattern described by eq 6 also indicates critical behavior, which evokes unusual properties in this domain. These unique features are explored in Supercritical Fluid (SCF) technology,^42–44^ with respect to a dramatic increase in selective solubility as the critical point is approached. The Whitney-Noyes and Kirkwood model relations describe the process.^43,44^ Their critical form, also showing the importance of dielectric constant, is given in ref 44. The “critical hyper-solubility” implies a tendency to deactivate local intermolecular bonds. Upon heating toward *T*_*m*_, a feedback mechanism appears, supporting the development of local defects covered by quasi-liquids, leading to granulation, the degree of which further increases. Simultaneously, the negative pressure in the nanolayers increases, but before reaching the extreme at T*, the system stability limit is reached earlier, resulting in implosion and subsequently supergranulation leading to abrupt liquefaction at *T*_*m*_.

Figure 2 shows also dielectric constant changes on cooling from the liquid phase, where solidification occurs at the freezing temperature *T*_*f*_ ≈ *T*_*m*_ – 8 K. Notable is a weak post-freezing effect and the pattern of changes in the solid state which coincides with the discussed below ∼*T*_*m*_ – 50 K with a hallmark suggesting a structural change near *T* = 247 ± 5 K.

To conclude the results of the given report, worth recalling is the only reference related to dielectric studies in the premelting domain of monohydric alcohols, namely ref 33, by Pocheć et al. (2020). It showed a permanent rise of *ε*′(*T* → *T*_*m*_, *f* = 4 MHz) in solid undecanol for *T*_*m*_ + 12 < *T* < *T*_*m*_. No scaling was considered.^33^ Reference 33 mainly focused on strong electric field related changes of Δ*ε*′ = *ε*′(*E*) – *ε*′(*E* → 0), linked to Nonlinear Dielectric Effect (NDE), defined as *NDE* = Δ*ε*/*E* ^2^ = (*ε*(*E*) – *ε*(*E* → 0))/*E* ^2^. In ref 33 the following scaling pattern obeying for all tested alcohols, from nonanol to dodecanol, was indicated Δ*ε*′ ∝ (*T*_*m*_ – *T*)^ϕ=–1^. The discussion heuristically suggested a link to a specificity of the plastic crystal rotator phase and critical phenomena, particularly exemplified in liquid critical binary mixtures.^33^ However, the singularity at *T*_*m*_, leading to Δ*ε*′(*T* → *T*_*m*_) → *∞*, was not explained or supported by the evidence. The question of whether the biasing dispersion impact in the premelting-effect domain, occurring in nonanol, also arises. The discussion of the premelting effect in monohydric alcohols was continued using FTIR (Fourier Transform Infrared Spectroscopy) insight.^34^ For nonanol, some liquid-type intramolecular motions for *T*_*m*_ – 4 K < *T* < *T*_*m*_ were noted.

In conclusion, the given report fills significant cognitive gaps related to the wide-temperature-range (∼190 K) dielectric constant behavior of nonanol, a representative of the monohydric alcohol family. Studies reveal unusual behavior in the liquid phase (eq 5b) and “forbidden” Mossotti Catastrophe behavior (eqs 6 and 7) in the premelting region of the solid phase. Notably, the latter agrees with the preliminary evidence for menthol,^45^ nitrobenzene,^46,47^ and liquid crystalline nanocolloids.^48^

This report, supported by the experimental evidence in refs 45–48 , suggests a new melting transition model with a possible inherent link to critical phenomena.
